# Hsp70 chaperone: a master player in protein homeostasis

**DOI:** 10.12688/f1000research.15528.1

**Published:** 2018-09-19

**Authors:** María Rosario Fernández-Fernández, José María Valpuesta

**Affiliations:** 1 Centro Nacional de Biotecnología (CNB-CSIC), Darwin 3, Campus de la Universidad Autónoma de Madrid, Cantoblanco, E-28049 Madrid, Spain

**Keywords:** Protein homeostasis, proteostasis, Hsp70, structural biology, cryoelectron microscopy

## Abstract

Protein homeostasis (proteostasis) is an essential pillar for correct cellular function. Impairments in proteostasis are encountered both in aging and in several human disease conditions. Molecular chaperones are important players for proteostasis; in particular, heat shock protein 70 (Hsp70) has an essential role in protein folding, disaggregation, and degradation. We have recently proposed a model for Hsp70 functioning as a “multiple socket”. In the model, Hsp70 provides a physical platform for the binding of client proteins, other chaperones, and cochaperones. The final fate of the client protein is dictated by the set of Hsp70 interactions that occur in a given cellular context. Obtaining structural information of the different Hsp70-based protein complexes will provide valuable knowledge to understand the functional mechanisms behind the master role of Hsp70 in proteostasis. We additionally evaluate some of the challenges for attaining high-resolution structures of such complexes.

## Introduction

Protein homeostasis (proteostasis)—the balance of protein synthesis, folding, trafficking, assembly, and degradation—is essential for correct cellular function, and cells have developed a number of strategies to control it under stress. Impaired proteostasis occurs in aging and is associated with several human diseases. Molecular chaperones are important players for proteostasis; in particular, the chaperone heat shock protein 70 (Hsp70) has an essential role in protein folding, disaggregation, and degradation ^1^. There are four major protein degradation pathways in mammalian cells: the ubiquitin-proteasome system (UPS) and three types of autophagy: macroautophagy, microautophagy, and chaperone-mediated autophagy (CMA). Hsp70 provides specificity for substrate selection to all of these degradation pathways in certain cellular contexts ^2^.

**Figure 1.**
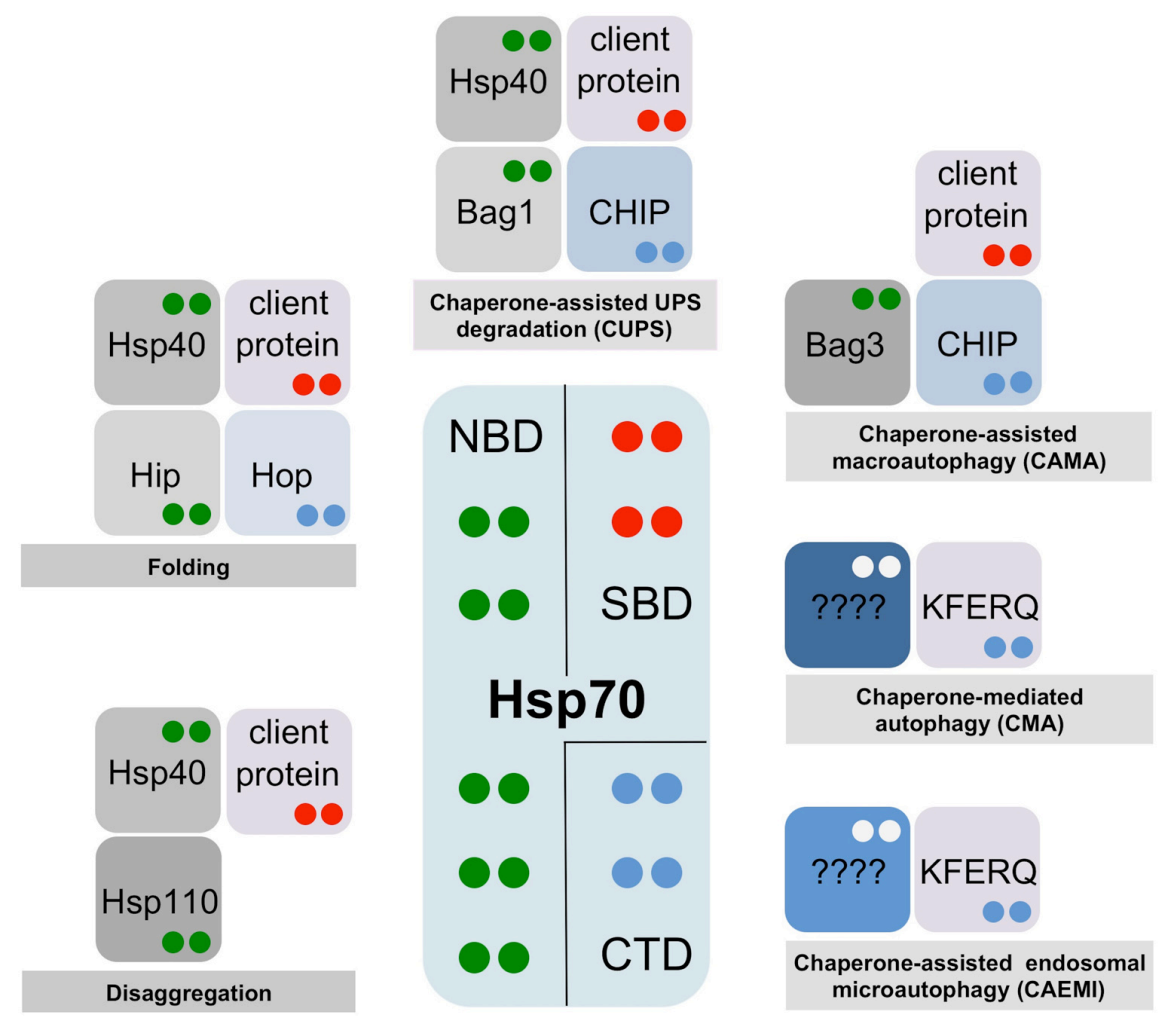
“Multiple socket” model for heat shock protein 70 (Hsp70) function in metazoan cells. The fate of a given Hsp70-bound protein (client proteins containing the classic hydrophobic motif or KFERQ-containing proteins) is dictated by the combination of Hsp70 interactors in a given cellular context. The color code of dots in interacting proteins indicates their Hsp70-binding domain—green: nucleotide-binding domain (NBD); red: substrate-binding domain (SBD); blue: C-terminal domain (CTD). Whereas classic client proteins bind to the Hsp70 SBD base domain, the KFERQ-containing proteins bind to a distinct, not yet determined, binding site ^12^.

Hsp70 has been referred to as the triage chaperone ^3^ as it determines the fate of client proteins. Cochaperone carboxyl terminus of Hsp70-interacting protein (CHIP) and Bag1 cochaperones have also been given this triage attribute independently ^4– 7^. Alternatively, we proposed that Hsp70 could be playing its master role in protein homeostasis in a more passive way, namely as a “multiple socket” that provides a physical platform for the binding of client proteins and for the interaction with different chaperones and cochaperones. The final combination of interactors dictates the fate of the Hsp70 client proteins ^2^ ( Figure 1).

It is important to note that whereas the prokaryotic system has a single Hsp70 (the archetypical DnaK), eukaryotes have an expanded number of genes that code for distinct Hsp70 isoforms, which function in different physiological conditions and subcellular locations ^2^. Consequently, it was reasonable to expect that the involvement of specific Hsp70 isoforms could also contribute to dictate the final fate of client proteins. Several reports have already confirmed this expectation; CMA appears to involve exclusively the constitutive Hsc70 isoform ^8^, human Hsp70 and Hsc70 differ considerably in their disaggregation potentials ^9^ and, surprisingly, while Hsc70 protects microtubule-associated protein tau (MAPT) from degradation, Hsp70 accelerates its clearance ^10^.

Although the mechanism of Hsp70 function has been extensively studied, certain aspects of its structure-function features are yet to be fully established. However, this information is critical for interfering with or modulating these pathways with therapeutic purposes in pathological conditions. Obtaining high-resolution structural information of the different Hsp70-based protein complexes will contribute considerably to understanding the molecular mechanisms in protein homeostasis. Here, we will discuss some of the challenges that Hsp70 and Hsp70-based complexes offer for structural biologists.

## The functional cycle and domain organization of Hsp70

**Figure 2.**
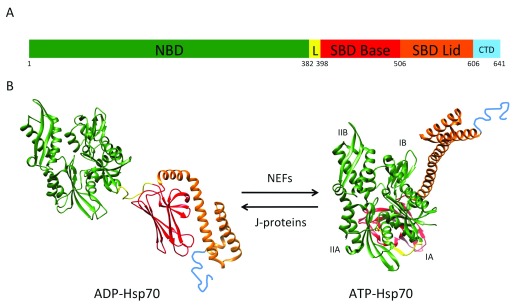
Heat shock protein 70 (Hsp70)-domain organization and atomic structures of functional conformations. ( **A**) Scheme of heat shock protein 70 (Hsp70) domain organization. The length of the segments is in scale with amino acid length. NBD: nucleotide-binding domain, aa 1-381; L: linker, aa 382-397; SBD Base: substrate-binding domain Base, aa 398-505; SBD Lid: substrate-binding domain Lid, aa 506-605; CTD: C-terminal domain, aa 606-641. Numeration is according to heat shock 70-kDa protein 1B *Homo sapiens* NP_005337.2. For simplicity, the numbering below the scheme shows only the first amino acid of each domain. ( **B**) Hsp70 atomic structures in the closed (PDB 2KHO) and open (PDB 4JNE) conformations. NBD subdomains are referred to as IB, IIB, IA, and IIA. The color code is as in ( **A**).

Hsp70 is a nanomachine that relies on the conformational changes fueled by ATP hydrolysis to assist protein folding, disaggregation, and degradation. Hsp70 is composed of two basic domains ( Figure 2A); the N-terminal, nucleotide-binding domain (NBD) (45 kDa) is a V-shaped structure composed of two subdomains (lobes) that enclose the ATP-binding site. The substrate-binding domain (SBD) (25 kDa) is also made of two subdomains: a beta-sheet domain (SBDβ) or Base and an alpha-helical (SBDα) or Lid domain. Hsp70 function involves allosteric interactions between the NBD and the SBD. The NBD drives conformational changes of the SBD, and substrate-induced conformational changes are transmitted from the SBD to the NBD interface ^11, 12^.

The SBD recognizes a motif present in most proteins on average every 30 to 40 residues; this short five-residue motif is enriched in hydrophobic amino acids and is flanked by regions with predominantly positively charged residues ^13^. These hydrophobic sequences are usually buried in the protein core and their accessibility signals a misfolded conformation. The SBD can adopt two conformations: open and closed ( Figure 2B). ATP binding to the NBD induces the SBD open conformation by separating the Base and Lid subdomains, a conformation of low affinity for the binding of client proteins. The hydrolysis of ATP drives the closed conformation; the structural rearrangements originate at the NBD and are transmitted via the linker to induce the SBD Base and Lid subdomains coming closer and trapping the client protein with high affinity. The flexible and conserved linker contacts the NBD in the open state and detaches from it in the closed conformation ( Figure 2B).

Basal ATPase activities of Hsp70 proteins are generally low and are increased by J-protein (Hsp40) cochaperones. J-proteins target client proteins to Hsp70 and at the same time stimulate ATPase activity and the efficient locking of the client protein ^14, 15^. Release of the client protein requires ADP dissociation, which is driven by the action of another set of cochaperones, the nucleotide exchange factors (NEFs) ^16^ ( Figure 2B). Thus, J-proteins induce ATP hydrolysis and potentiate the closed conformation, whereas NEFs facilitate ADP/ATP exchange and consequently the open conformation.

## Hsp70: the “multiple socket” of protein homeostasis

### Hsp70 in protein folding

Anfinsen reported that the primary sequence of a protein determines its native fold ^17^. Later studies provided evidence that some proteins, especially large ones, require the coordinated contribution of different molecular chaperones and cochaperones for efficient folding. Hsp70 and Hsp90 chaperones cooperate in eukarya to coordinate the folding and maturation of key regulatory client proteins ^18^. Hsp90 is a dimeric chaperone that acts downstream of Hsp70. Hsp90 binds client proteins through an extended interface that enables a large number of low-affinity contacts. These chaperones are assisted by several cochaperones like Hsp40s, which have an essential role in targeting substrates to Hsp70 ( Figure 1), or Hsp70/Hsp90-organizing protein (Hop) that facilitates the transferring of substrates from Hsp70 to Hsp90 ^19^.

Interestingly, a very recent report reconsiders the precise function of some of these molecular chaperones in protein folding ^20^. Morán Luengo *et al*. ^20^ provided evidence that Hsp70 and Hsp90 may not work as foldases themselves but instead prepare proteins for spontaneous, productive folding as stated by Anfinsen. These results are in favor of the previous hypothesis of Goloubinoff *et al*., who proposed that chaperones are not foldases but act catalytically to unfold misfolded polypeptides that then will spontaneously refold ^21^.

### Hsp70 in protein disaggregation

Protein disaggregation is an essential event in protein homeostasis in different physiological and pathological cellular circumstances. Hsp70 plays a central role in disaggregation processes and, as discussed below, seemingly by different mechanisms throughout evolution.

Hsp70 recruits and activates Hsp100 disaggregases ^22^ in bacteria, fungi, and plants to extract aggregated proteins ^23^. Metazoans lack an Hsp100 disaggregase; in this case, specific J-proteins and Hsp110 cochaperones (a type of NEF) cooperate with Hsp70 to perform disaggregation in the absence of a dedicated disaggregase ( Figure 1). In both scenarios, small heat shock proteins are essential players in the sequestration of misfolded proteins and impact the final downstream fate of the aggregated protein ^24^.

### Hsp70 in protein degradation

For many years, protein degradation attracted considerably less attention than protein synthesis and folding. Later work showed that protein degradation is not only important for the clearance of misfolded or damaged proteins but also relevant for normal cellular function.

***Chaperone-assisted ubiquitin-proteasome system degradation.*** The UPS tags proteins with one or more ubiquitin molecules, facilitating their entry into the proteasome for degradation ^25^. The chaperone-assisted ubiquitin-proteasome system (CUPS) ^2^ involves the ubiquitination of a chaperone-bound client protein and its subsequent sorting to the proteasome.

In metazoan cells, CUPS involves the direct docking of the ubiquitin ligase CHIP to the Hsp70 C-terminus. This interaction favors the ubiquitination of Hsp70-bound client proteins. In CUPS, the NEF Bag1 coordinates the release of the misfolded protein from Hsp70 with proteasome interactions ^26– 28^. Bag1 interacts with the proteasome through a ubiquitin-like (UBL) domain, and ubiquitylation of Bag1 by the E3 ligase CHIP enhances this association ^28, 29^. Thus, Bag1 releases the Hsp70 client proteins near the proteasome, facilitating their degradation ^28^ ( Figure 1).

In yeast, Hsp70 and Hsp110 have recently been shown to cooperate in the delivery of ubiquitin-dependent and -independent client proteins to the 26S proteasome. Hsp110 associates with the 19S regulatory particle of the proteasome and interacts with Hsp70 to facilitate the delivery of Hsp70 client proteins ^30^. For organisms where Hsp110 and Bag1 coexist, it will be interesting to explore whether both proteins act indistinguishably in CUPS or whether they provide any kind of specificity to the degradation pathway.

***Chaperone-mediated autophagy.*** CMA involves the specific lysosomal degradation of intracellular proteins ^8^. In CMA, the Hsc70 isoform binds to proteins at sequences biochemically similar to KFERQ ^31^. This amino acid sequence is unrelated to the hydrophobic peptide motif of Hsp70 classic client proteins ^13^. LAMP2A was identified as the CMA receptor at the lysosomal membrane ^32^. LAMP2A is a single-span 45-kDa membrane protein with a 12–amino acid cytosolic domain and a large luminal domain. The short cytosolic tail is essential in the selection of CMA substrates.

The precise mechanistic aspects of the CMA pathway are still not fully understood. The binding of Hsc70 to target proteins is proposed either to convert them into transport-competent structures or to facilitate their interaction with the lysosomal receptor ^31^. In fact, more recent results suggested that client proteins are selected for CMA only if they simultaneously bind to Hsc70 (via the KFERQ motif) and to the LAMP2A cytosolic tail ( Figure 1). This implies that the interaction sites for client proteins on Hsc70 and LAMP2A are distinct ^33^.

Binding of CMA client proteins to LAMP2A induces the formation of a CMA translocation complex, composed of several copies of LAMP2A and other yet-undetermined proteins ^34^. The final intake of cytosolic proteins for degradation by proteases into the lysosome requires the action of an intralysosomal form of Hsc70 ^35, 36^.

Several experimental approaches indicated that Hsc70 forms complexes at the lysosomal membrane with other molecular chaperones and cochaperones ^34, 37, 38^. However, the precise nature of these Hsc70-protein interactions and their mechanistic role in CMA is still to be determined.

***Chaperone-assisted endosomal microautophagy.*** Microautophagy involves the direct entrapment of cytosolic components by the invagination of the lysosomal membrane and degradation of the cargo-containing vesicles. Microautophagy has been well characterized in the yeast vacuole ^39– 41^ but remains poorly understood in mammalian cells.

There is a specific form of microautophagy that occurs at late endosomes (chaperone-assisted endosomal microautophagy, or CAEMI) in mammalian cells. During the formation of multivesicular bodies, cytosolic proteins are trapped in late endosomal vesicles. The process relies on electrostatic interactions of Hsc70 with endosomal acidic phospholipids ^42, 43^. Several lysine residues at the Hsc70 Lid domain have been identified as the interface interacting with endosomal phosphatidylserine ^44^. As in CMA, Hsc70 selects client proteins for CAEMI through the recognition of the KFERQ peptide motif ( Figure 1), and the process seems to require the oligomerization of Hsc70 ^45^. However, the mechanistic details of CAEMI are not yet fully understood. The fact that CAEMI and CMA share many features, including the specific involvement of the Hsc70 isoform or the selectivity for substrates with the KFERQ motif, has precluded a more extensive knowledge of both pathways.

***Chaperone-assisted macroautophagy.*** Macroautophagy was long considered the non-specific bulk degradation of cytosolic components after their enclosure by the autophagosome, a double-membrane vesicle that further fuses with the lysosome for cargo degradation ^46^. However, it was later shown that macroautophagy also employs mechanisms that target for specific substrates ^47^, and the disposal of aggregate-prone proteins linked to human diseases has attracted considerable interest ^48^.

Hsp70 nucleates a number of interactions that are basic for the specific delivery of ubiquitinated client proteins to the forming phagophore. Hsp70 interacts with the cochaperone Bag3 ( Figure 1), a molecular linker that coordinates the action of Hsp70 and the small heat shock protein, HspB8. The three proteins form a ternary complex with Bag3 linking Hsp70 and HspB8 ^49^. Additionally, the ubiquitin ligase CHIP associates with the ternary complex through the Hsp70 C-terminal domain ^50– 53^. The preloading complex composed of the ubiquitinated client protein, CHIP, Hsp70, Bag3, and HspB8 is recruited by the ubiquitin-binding protein p62/SQSTM1, which binds simultaneously to ubiquitinated client proteins and LC3 ^54^. The lipidated form of LC3 (LC3-II) associates with the phagophore membrane. The simultaneous binding to the loading complex and the membrane could trigger enclosure of the aggregated protein within the autophagosome ^55^.

## Future perspectives: the structural challenge

Despite the central role of Hsp70 in protein homeostasis, structural work of Hsp70 and Hsp70-complexes has been limited by the intrinsic characteristics of the protein.

A substantial number of Hsp70 family members are known to oligomerize to form dimers and higher-order oligomers in a concentration-dependent manner. The ADP-bound and nucleotide-free forms of Hsp70 are more prone to oligomerize *in vitro*, and the addition of ATP, client protein binding, or the presence of some cochaperones seems to revert the oligomerization ^56– 58^. Post-translational modifications also stabilize the dimerization of Hsp70 ^59^. The physiological relevance of the oligomerization is still unclear, and it has been suggested that this could be a mechanism for regulating the availability of active Hsp70 monomers and for the control of the client protein binding. There are already reports confirming that Hsp70 oligomers are functionally distinct from monomers ^60^; however, further studies are necessary to fully understand the relevance of these differences. Hsp70 oligomerization seems to involve the interdomain linker and the SBD, precisely the Lid domain ^61^, and occurs in the micromolar range, which seems likely to be the physiological concentration range of Hsp70 ^62, 63^.

Interestingly, in the majority of constructs used for defining nuclear magnetic resonance (NMR) and crystal structures of the full-length protein or isolated Hsp70 domains, most of the residues of the Lid domain have been deleted to avoid the tendency to oligomerize at the high concentrations required for such structural analysis. However, the Lid domain is known to have a prominent role in the binding of the specific client proteins ^64^, and the most C-terminal sequence of Hsp70 is important for the binding of cochaperones such as CHIP or Hop ^65, 66^. This implies that structural studies aiming to resolve the mechanisms of the fine-tuned Hsp70 function should not disregard these important Hsp70 domains.

The interdomain linker that tethers the NBD and the SBD is well structured and participates in the interdomain interface in the ATP-bound Hsp70 (the state of low affinity for client proteins) ( Figure 2B). However, in the ADP-bound state, the interdomain linker is flexible. Molecular dynamics simulations have shown that the interdomain linker adopts many conformations but behaves basically as three relatively ordered segments connected by hinges ^67^. Consequently, there are a limited number of distances and orientations between the NBD and the SBD. NMR analysis of the ADP-bound DnaK also showed the restrictions imposed by the linker to the relative orientations of the NBD and SBD. The interdomain paramagnetic relaxation data were consistent with a restriction of the NBD and SBD relative orientations to a 35° cone ^68^. Eukaryotic Hsp70 interdomain linkers are generally longer than prokaryotic ones ^67^. The flexible nature of the linker implies that biologically interesting Hsp70-complexes (that is, those that include client proteins) are particularly flexible, precluding high-resolution structural determination by x-ray crystallography.

In this context, cryoelectron microscopy (cryoEM) offers advantages over other structural techniques. The amount of protein needed for the analysis of Hsp70-based macromolecular complexes is considerably lower than for other structural techniques. For example, it works well in the low micromolar range where the effects of Hsp70 oligomerization are still negligible. CryoEM is also more tolerant to protein structural flexibility and to the presence of different macromolecular populations. The advances that cryoEM has experienced in recent years (new generation of electron detectors, phase plates, and image-processing tools) have maximized the advantages of cyroEM over other structural techniques ^69– 71^. New image-processing tools facilitate the classification of data sets coming from heterogenous samples. A very impressive example is the work with the 80S ribosome in complex with the eIF5B factor. Although the intact complex represented only 3% of all the recorded particle images, classification tools allowed a 6.6-Å map to be obtained from the analysis of 5000 particles ^72^. The flexibility of macromolecular complexes precludes their analysis by x-ray crystallography but also poses a challenge for cryoEM analysis. Interestingly, new imaging tools are being developed to improve the local resolution of structured regions within a flexible macromolecular complex. A recent example is multi-body refinement, a new tool that models flexible complexes as a defined number of rigid bodies that move independently from one another ^73^. It will be interesting to explore the potential of these advances in the detailed structural characterization of Hsp70 protein complexes.

Future structural studies by cryoEM could also benefit from molecular tools that have been explored in other structural approaches. Nanobodies are a particularly attractive example; they are small single-domain fragments that preserve the antigen-binding capacity of the heavy chain–only antibodies that naturally occur in camelids ^74^. There are many examples of the successful use of nanobodies in x-ray crystallography ^75, 76^ and NMR ^77^ structural studies. The binding of nanobodies can contribute to increase the solubility of target proteins and to stabilize specific protein conformations. These are useful properties when solving the structure of flexible multidomain proteins.

Finally, an interesting point to consider when analyzing Hsp70-protein complexes is that many studies are performed with a certain Hsp70 family member and that the conclusions are often extrapolated to the entire Hsp70 family. This is based on the fact that Hsp70s are among the most conserved proteins in evolution ^78^. However, we should take into account the evidence of different Hsp70 isoforms having different fitness in specific functions ^8– 10^. These examples support the need to explore in further detail the contribution of isoforms to the diversity of Hsp70 function, an area that has otherwise been understudied.
